# Diagnostic Accuracy of Non-Imaging and Ultrasound-Based Assessment of Hepatic Steatosis Using Controlled Attenuation Parameter (CAP) as Reference

**DOI:** 10.3390/jcm10071507

**Published:** 2021-04-04

**Authors:** Katarzyna Kozłowska-Petriczko, Ewa Wunsch, Jan Petriczko, Wing-Kin Syn, Piotr Milkiewicz

**Affiliations:** 1 Translational Medicine Group, Pomeranian Medical University, 70-204 Szczecin, Poland; kasia-petriczko@outlook.com (K.K.-P.); p.milkiewicz@wp.pl (P.M.); 2 Department of Gastroenterology and Internal Medicine, SPWSZ Hospital, 71-455 Szczecin, Poland; 3 Department of Plastic, Endocrine and General Surgery, Pomeranian Medical University, 70-204 Szczecin, Poland; jan.petriczko@gmail.com; 4 Division of Gastroenterology and Hepatology, Medical University of South Carolina, Charleston, SC 29425, USA; synw@musc.edu; 5 Liver and Internal Medicine Unit, Medical University of Warsaw, 02-097 Warsaw, Poland

**Keywords:** liver steatosis, Hamaguchi’s score, hepatorenal index, biomarkers, diagnostics

## Abstract

Background & Aims: In view of the limited reliability of biopsies in the assessment of liver fat, a non-invasive, trustworthy, and more accessible method estimating a degree of steatosis is urgently needed. While the controlled attenuation parameter (CAP) is used to quantify hepatic fat, its availability in routine practice is limited. Therefore, the aim of this study was to compare the diagnostic accuracy of biomarker- and ultrasound-based techniques for the diagnosis and grading of hepatic steatosis. Methods: This was a prospective study of 167 adults with and without non-alcoholic fatty liver disease. As measured against CAP, we assessed Hamaguchi’s score and the hepatorenal index (HRI), and the following biochemical measures: the fatty liver index, hepatic steatosis index, and lipid accumulation product scores during a single out-patient visit. Area under the receiver operating curve (AUROC) analyses were used to evaluate the diagnostic accuracy of each test and to calculate optimal thresholds for the ultrasound techniques. Results: All non-invasive methods displayed high accuracy in detecting steatosis (mean AUC value ≥ 0.90), with Hamaguchi’s score and the HRI being the most precise. These two tests also had the highest sensitivity and specificity (82.2% and 100%; 86.9% and 94.8%, respectively). We propose new thresholds for Hamaguchi’s score and HRI for hepatic steatosis grading, indicated by optimal sensitivity and specificity. Conclusions: Ultrasound-based techniques are the most accurate for assessing liver steatosis compared to other non-invasive tests. Given the accessibility of ultrasonography, this finding is of practical importance for the assessment of liver steatosis in clinical settings.

## 1. Introduction

Nonalcoholic fatty liver disease (NAFLD) has become the most common liver disorder in Western countries, and its global prevalence is estimated at 25.2% [1]. NAFLD may progress from simple steatosis to nonalcoholic steatohepatitis (NASH) and then to liver cirrhosis, which is associated with an increased risk of hepatocellular carcinoma and other cirrhosis-related complications [1]. The main risk factors for NAFLD include components of the metabolic syndrome such as diabetes, obesity, dyslipidaemia, and hypertension, and it has been suggested that patients with these co-morbidities should be actively screened for NAFLD, even if their liver enzymes are within normal ranges [2,3,4]. Currently, effective screening for NAFLD in clinical practice is hindered by a lack of clear guidelines concerning non-invasive diagnostic tools. Liver biopsy (LB) remains the gold standard for the diagnosis of NASH and assessment of fibrosis, but not for hepatosteatosis [1]. Furthermore, biopsies have known disadvantages that include a risk of complications due to the invasive nature of the procedure, sampling variability because of the small size of tissue obtained, and the heterogeneous distribution of histological changes in liver parenchyma [5]. Moreover, due to the extent of the condition (e.g., 80 million Americans are affected by NAFLD [6]), routine LBs to confirm NAFLD may be unwarranted, and could even be considered unethical. Undoubtedly, LB remains the only tool available to confirm NASH, even though the prevalence of NASH among NAFLD patients is estimated at 1.5–6.5% [7]. Therefore, LBs should only be considered in those patients at high risk for the progressive type of the disease.

To find a new standard for liver steatosis screening, and to replace the use of LBs in most patients at low risk for NASH, non-invasive diagnostic methods (biomarkers and imaging-based techniques) are required. The controlled attenuation parameter (CAP) (FibroScan system; Echosens, Paris, France) integrated with Fibroscan—a modality estimating liver steatosis and fibrosis using transient elastography—remains a reliable and one of the most-studied quantitative tools that has been validated against LBs [8,9,10,11,12]. CAP calculates the attenuation of an ultrasound beam traversing the liver tissue. It is observer-independent and evaluates an area 100 times larger than an LB. CAP is considered to be an accurate tool for the diagnosis and staging of hepatic steatosis, with mean area under the receiver operating characteristic (AUROC) values for the diagnosis of mild, moderate, and severe steatosis of 0.9, 0.8, and 0.7, respectively [8]. The inter-observer reproducibility of the technique is high, with a concordance correlation coefficient of 0.82 (0.78–0.85). However, there are studies on the inter-observer concordance in CAP, which have shown discrepancies of results obtained by two observers of up to 20dB/m [13]. Moreover, CAP measurement results can be influenced by metabolic syndrome or a high body mass index (BMI). In extremely obese patients, measurement failure can occur. To improve these limitations, the XL probe was developed [13].

However, the equipment needed to undertake this assessment is usually unavailable in non-hepatological centres, such as in clinics for general practitioners or peripheral hospitals. During routine practice, as the European Association for the Study of the Liver guidelines recommend [14], liver steatosis is typically screened using an abdominal ultrasound, despite its limitations, which include subjective evaluations, operator dependency, and the ability to only recognize fatty liver infiltration that is greater than 20% in histology [13]. To enhance objectivity, Hamaguchi et al. [15] proposed an alternative, semi-quantitative, ultrasound-based steatosis assessment score that has 91.7% sensitivity and 100% specificity. Alternatively, the hepatorenal index (HRI) designed by Webb et al. [16] is another quantitative, ultrasound-based, hepatic-steatosis measure that correlates with LBs and has an AUROC of over 0.9 for all steatosis grades. To identify simpler and cost-effective approaches for the diagnosis of NAFLD, several scores based on easily measurable biochemical and clinical parameters, such as the fatty liver index (FLI), [17] hepatic steatosis index (HSI) [18], and lipid accumulation product (LAP) [19] have also been developed. All of these non-invasive tools are potentially useful screening methods for clinical practice; however, the choice of an optimal screening modality as part of a daily clinical routine is made difficult by a lack of comparative studies that assess their accuracy, as well as there being no clear guidelines for clinicians.

Therefore, the aim of this study was to undertake a comparative assessment of the diagnostic accuracy for the detection and quantification of hepatic steatosis using both ultrasound-based and biochemical techniques. Specifically, we investigated two validated ultrasound methods (Hamaguchi’s score and the HRI) and three biochemical panels (FLI, HSI and LAP) using CAP as the reference method.

## 2. Materials and Methods

### 2.1. Study Population and Design

A total of 177 adult consecutive patients were prospectively recruited between March 2018 and February 2020 in a single out-patient centre located in Szczecin, Poland. The study was designed following the Standards for Reporting of Diagnostic Accuracy (STARD) guidelines (Table S1). The study protocol was approved by the local ethics committee (Pomeranian Medical University, Szczecin, Poland; the approval number: KB-0012/08/18) and conformed to the ethical guidelines of the 1975 Helsinki declaration, and patients were enrolled after giving their written informed consent. Inclusion criteria were as follows: 18 years of age, willing and able to complete all procedures described in study protocol, and who had given written informed consent. Men who ever consumed more than 30 g of alcohol per day and women who ever consumed more than 20 g of alcohol per day, as well as patients with potential secondary causes of hepatic steatosis (including nutritional, infectious, iatrogenic etiology), those with known chronic liver diseases, including viral hepatitis (positive hepatitis B surface antigens or anti-hepatitis C virus antibodies), autoimmune, genetic, or acquired disorders, patients with major systemic illnesses, and individuals with chronic kidney disease were excluded.

**Figure 1:**
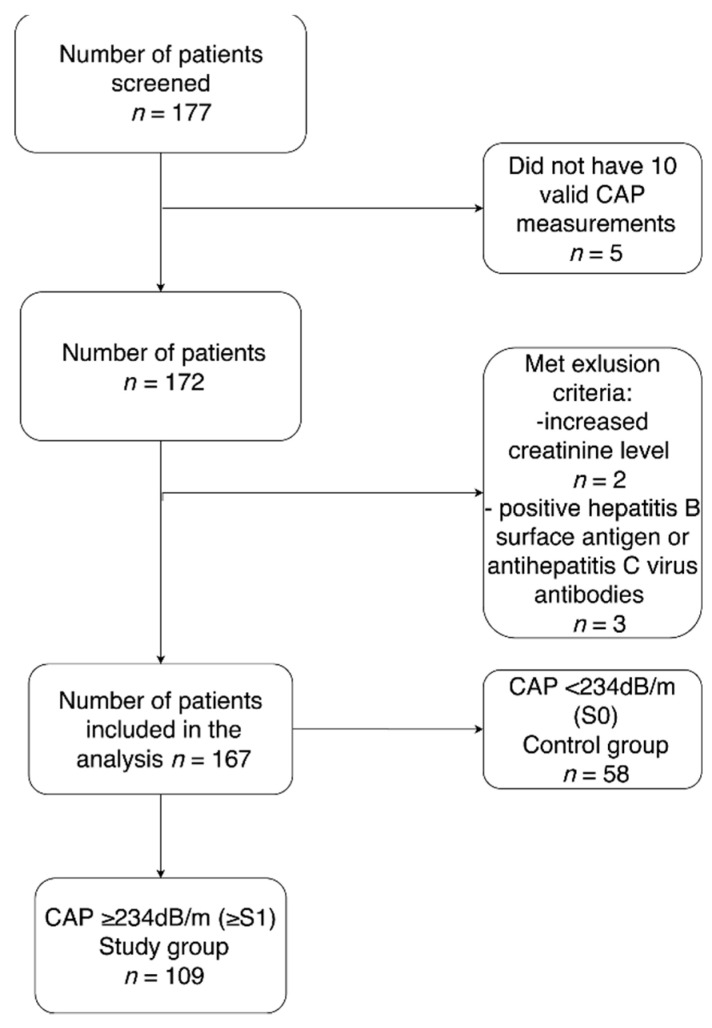
The study flow chart.

Among the 177 patients initially screened, 167 were included in the final data analysis (Figure 1). Patients were divided into two groups according to their CAP results using the cut-off values of Karlas et al. [10]. Fifty-eight participants without hepatic steatosis (S0) were included as a control group, and 109 patients with CAP-confirmed liver steatosis (≥S1) constituted the study group. Participant characteristics are presented in Table 1. The majority of patients were female (61.7%), the average age was 53 ± 12 years. Average BMI was 28.6 ± 5.0 kg/m^2^, and 9.5% of participants suffered from diabetes, while 34.5% had hypertension.

### 2.2. Clinical Assessment

Demographic and anthropometric measurements, including body mass index (BMI), waist and hip circumferences, questionnaires regarding medical history, and current and past alcohol ingestion were obtained. The presence and severity of steatosis was evaluated using CAP as the reference method. CAP and abdominal ultrasound for obtaining Hamaguchi’s score and the HRI were performed during the same appointment by the same trained operator (KKP) after patients had fasted for at least 4 h. Fasting blood samples were collected to obtain biochemical data.

### 2.3. CAP Measurements

Liver stiffness and CAP measurements were performed using FibroScan^®^ (Echosens, Paris, France). Measurements were obtained using both M (3.5 MHz) and XL (2.5 MHz) probes, depending on skin-to-liver capsule distance (≤25 mm or >25 mm), and the probe selection was guided by an integrated tool. Steatosis grades were established using the following cut-off values for low-, intermediate-, and high-grade steatosis (S1, S2, S3): 234, 269, and 301 dB/m, respectively [10]. These values were preferably integrated in FibroScan cut-offs for quantifying NAFLD.

### 2.4. Ultrasound Examination: Hamaguchi’s Score and the HRI

The hepatic ultrasound was performed using a high-resolution B-mode tomographic ultrasound system (Aixplorer, SuperSonic Imagine, Aix-en-Provence, France) with a convex SC6-1 probe in abdominal mode. Patients were examined in the dorsal position when Hamaguchi’s score and the HRI were calculated. Hamaguchi’s ultrasound score uses a number of variables to assess liver steatosis: hepatorenal echo contrast, liver parenchyma brightness, vessel blurring, and attenuation depth. Hepatic steatosis is defined by a score ≥2 and moderate/severe steatosis by score ≥4 [15]. The HRI is the ratio of the average liver parenchyma to renal cortex brightness in a B-Mode sonogram. A ratio below 1.49 signified no steatosis. Low, intermediate, and high grades were diagnosed when the HRI ranged between 1.49–1.85, 1.86–2.22, or 2.23 and greater, respectively [16].

Technical details of non-invasive techniques applied in the study are presented in Supplementary Material 1.

### 2.5. Fatty Liver Disease Algorithms

Three liver steatosis algorithms: the FLI, HSI, and LAP were calculated using clinical, anthropometric, and laboratory data, obtained at the same appointment as the ultrasound examination according to the following formulas:

FLI = (e0.953 × loge (triglycerides) + 0.139 × BMI + 0.718 × loge (GGT) + 0.053 × waistcircumference − 15.745)/(1 + e0.953 × loge (triglycerides) + 0.139 × BMI + 0.718 × loge (GGT) + 0.053 × waistcircumference − 15.745) × 100.

FLI scores range from 0 to 100 and have been previously validated against ultrasound methods for liver steatosis detection, with a sensitivity of 0.61 and specificity of 0.86 for a cut-off value of 60 [17].

HIS = 8 × ALT/AST ratio + BMI (+2, if diabetes mellitus; +2, if female). A result of 36 or greater is considered high-risk for liver steatosis [18].

LAP = (waist circumference − 65) × triglycerides in men and (waist circumference − 58) × triglycerides in women. A LAP result of 23 in women and 30.5 in men is considered at risk for liver steatosis [19].

### 2.6. Statistical Analysis

The Kolmogorov–Smirnov test was used to assess the distribution of variables. Qualitative variables are presented as counts and percentages. Continuous variables are shown as mean ± standard deviation (SD) or as medians with interquartile ranges (IQRs). Parametric tests (Student’s *t*-test and ANOVA) were used for the assessment of differences between numerical variables with normal distributions, and non-parametric tests (Mann-Whitney or Kruskal-Wallis tests) were used for variables with non-normal distributions. Pearson’s coefficient (ρ) was used to evaluate the association between two continuous variables. To evaluate the effectiveness of different scores in predicting (S > 0) and grading (S1, S2, S3) NAFLD, the receiver operating characteristic (ROC) curves, and sensitivity and specificity was constructed using CAP as a reference. The AUROCs, with 95% CIs, were recorded and used to establish new optimal thresholds for detecting and grading liver steatosis. The Spearman’s rank coefficient was calculated to analyse inter-rater reliability between ordinal diagnostic scales and liver steatosis severity, as established by CAP. Multi-variate forward and backward stepwise logistic regression analyses (using CAP as the dependent variable and the scores, and the biochemical and anthropometric parameters as the independent variables) were used to evaluate algorithms that strongly correlated (R) with hepatic steatosis. Two-sided *p* values < 0.05 were considered significant. All statistical analyses were performed using STATA 11 software (StataCorp, College Station, TX, USA).

## 3. Results

### 3.1. Correlation between CAP and Non-Invasive Indexes

**Figure 2:**
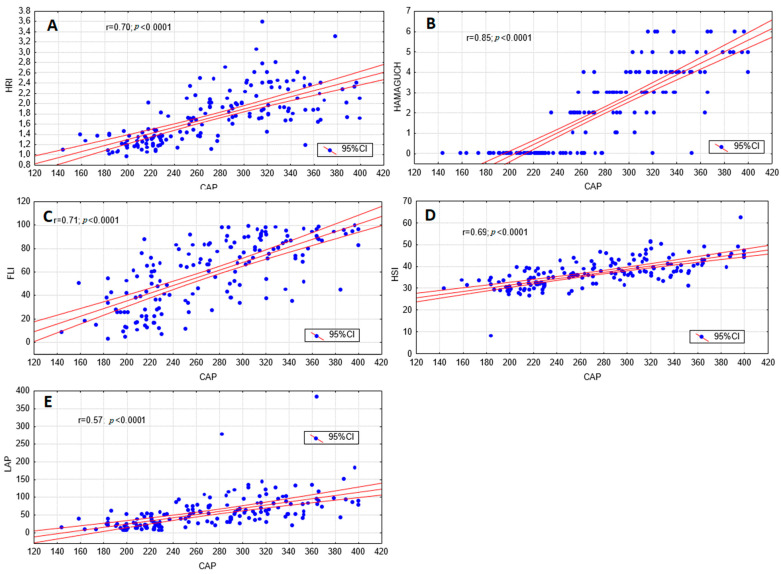
Correlation between controlled attenuation parameter (CAP) and non-invasive measures of steatosis. Correlations between liver steatosis diagnostic methods according to CAP and the hepatorenal index (HRI) (**A**), Hamaguchi’s score (**B**), fatty liver index (FLI) (**C**), hepatic steatosis index (HSI), (**D**) and lipid accumulation product (LAP) (**E**). Pearson’s coefficient (ρ) was used to evaluate the association between two continuous variables.

**Figure 3:**
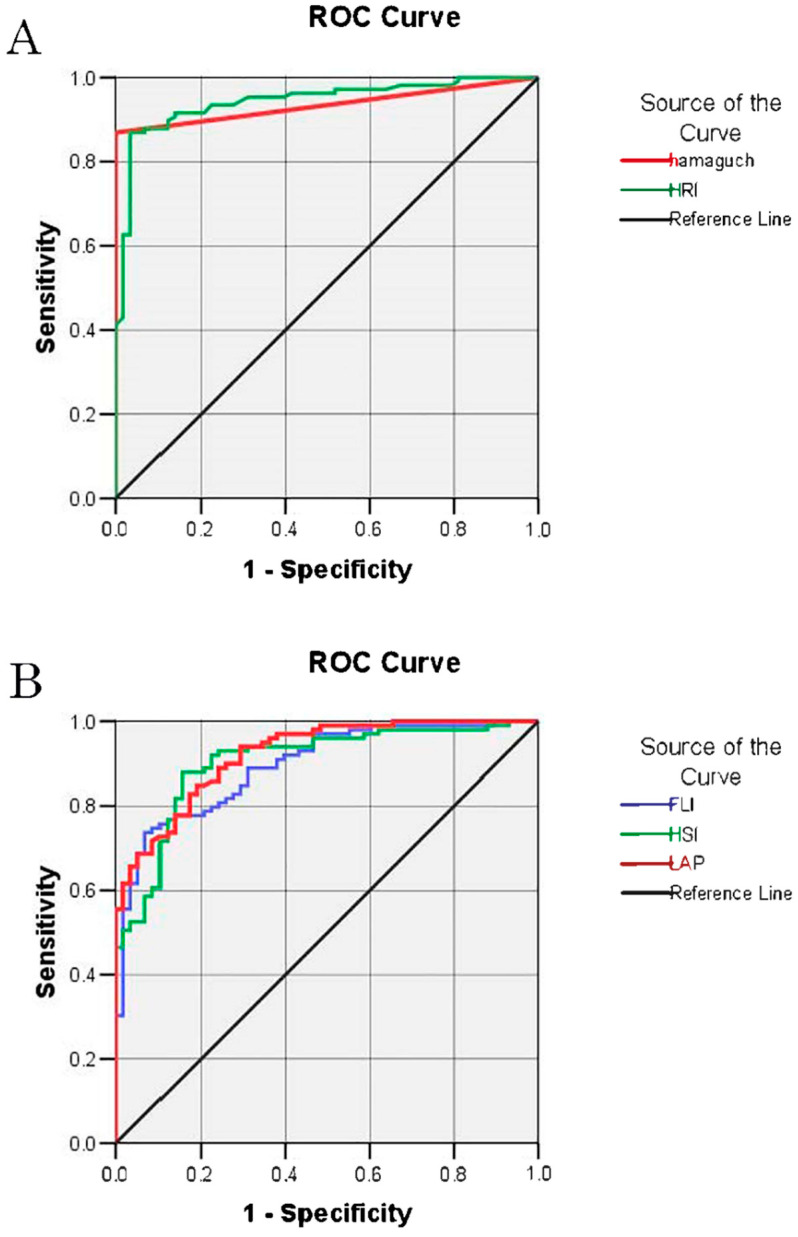
The receiver-operating characteristic (ROC) curve for the detection of non-alcoholic fatty liver disease (CAP > 234) for ultrasound-based (A) and non-imaging (B) methods. Abbreviations: CAP, controlled attenuation parameter; HRI, hepatorenal index; HSI, hepatic steatosis index; FLI, fatty liver index; LAP, lipid accumulation product; ROC, receiver operating characteristic.

**Figure 4:**
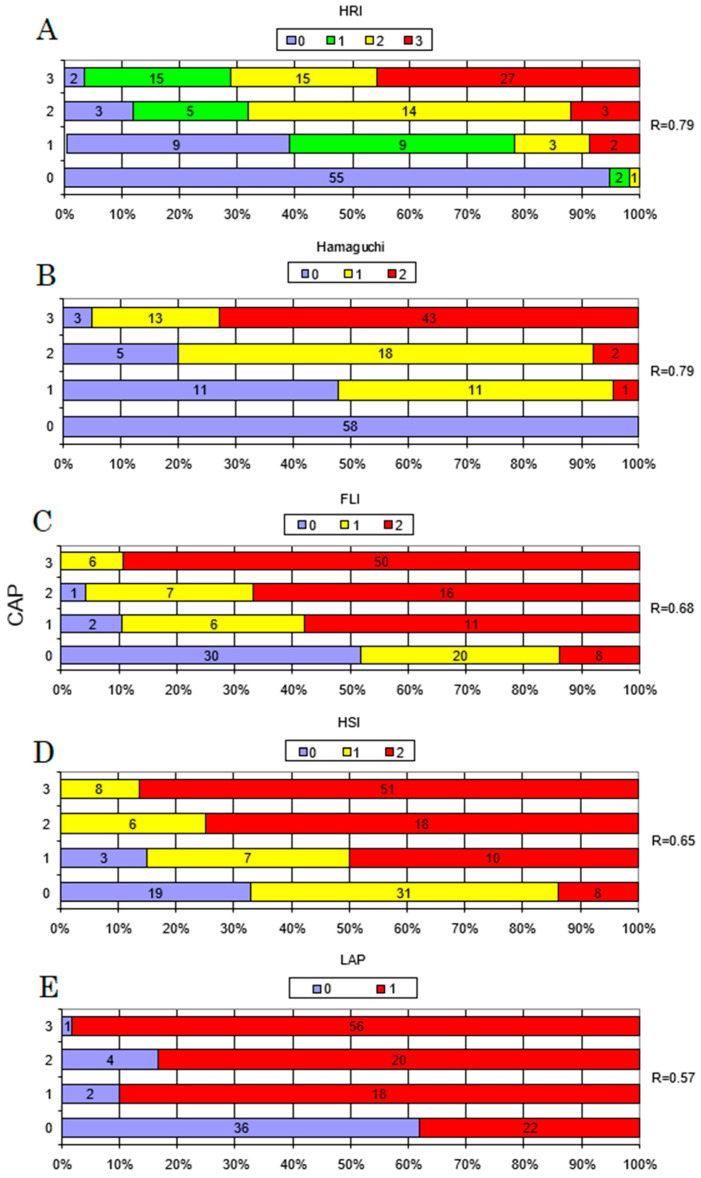
Discrepancies between controlled attenuation parameter (CAP) steatosis grades and hepatorenal index (HRI), Hamaguchi’s score, fatty liver index (FLI), hepatic steatosis index (HSI), and lipid accumulation product (LAP) steatosis grades. Comparison between classification of liver steatosis according to CAP and HRI (**A**), Hamaguchi’s score (**B**), FLI (**C**), HSI (**D**), and LAP (**E**). The Spearman’s rank (R) coefficient was calculated to analyse the inter-rater reliability between ordinal diagnostic scales and liver steatosis severity established by CAP. CAP grades S1–S3 are described according to Karlas et al. [10], with cut-off values of 234, 269, and 301 dB/m, respectively.

All tests displayed acceptable accuracy in discriminating the presence of steatosis as defined by CAP, and all were significantly correlated with hepatic fat content, as measured with CAP (Figure 2). The diagnostic performance data for the detection of steatosis (CAP ≥ 234 dB/m) are presented in Table 2, with the ROC curves shown in Figure 3. The sensitivity and specificity of the methods were measured based on the AUROC results and are listed in Table 2. The highest sensitivity was achieved by the LAP and the highest specificity by Hamaguchi’s score. As presented in Figure 4, the highest inter-rater reliability (R) in comparison to CAP was the ultrasound-based grading scores (R = 0.79), with biochemical algorithms having slightly lower values. All participants from the control group (S0) achieved 0 points in Hamaguchi’s score, and 95% had HRIs <1.49. Forty-three patients (73%) out of these diagnosed with S3 steatosis had 4 or more points using Hamaguchi’s score. When comparing Hamaguchi and the HRI versus CAP for the identification of steatosis of any severity (≥S1), these methods were able to correctly identify 82% and 87% of patients using thresholds of 2 and 1.49 points, respectively.

### 3.2. Optimal Thresholds

Based on the AUROC results, new optimal thresholds for HRI and Hamaguchi’s score, according to CAP, were calculated and listed in Table 3. The estimated cut-off values reached high sensitivity and specificity values for steatosis grading. The only value with a sensitivity lower than 70% was the HRI threshold for detecting severe steatosis.

### 3.3. Optimal Steatosis Prediction Model

Based on the multi-variate forward and backward stepwise logistic regression analysis, different imaging, biochemical and anthropometric parameters and scores were evaluated to find a combination model that strongly correlated with hepatic steatosis (Table 4). The application of Hamaguchi’s score together with the HSI achieved a correlation rate of 0.87. An even better result was found for the diagnostic model that relied on Hamaguchi’s score, BMI, GGTP, and ferritin levels, with a correlation rate of 0.89.

## 4. Discussion

### 4.1. Main Findings

In this study, we performed a comparative analysis of ultrasound-based and biochemical techniques for the non-invasive assessment of liver steatosis with CAP as a reference modality. We report that all tests attained high accuracy in detecting steatosis in comparison to CAP. Furthermore, we have demonstrated that ultrasound-based techniques (Hamaguchi’s score and the HRI) are more accurate than biochemical indexes. Of the biochemical panels, the FLI reached the highest accuracy level for detecting NAFLD. Our results are similar to those reported in previous studies that have validated these tests against LBs or magnetic resonance imaging proton density fat fraction (MRI-PDFF). We have also proposed threshold values for ultrasound methods that allow for the diagnosis and grading of liver steatosis.

Recent American Association for the Study of Liver Diseases Practice Guidelines on NAFLD do not recommend LBs in patients with NAFLD unless there is a strong suspicion of advanced fibrosis [1]. Therefore, a non-invasive measurement of liver steatosis plays a crucial role in the assessment of this pathology. In routine practice, liver steatosis is typically diagnosed with an ultrasound, an easily accessible and inexpensive modality. Nonetheless, this method is subjective and imprecise in follow-ups. Therefore, this study attempted to validate two, simple-to-perform, alternative ultrasound-based methods and three well-known biochemical panels for the evaluation of liver steatosis. Moreover, we investigated whether these tests could also be used for the quantification of steatosis.

#### 4.1.1. Ultrasound-Based Techniques

Hernaez et al. [20] published a meta-analysis based on forty-nine studies (4720 participants) investigating the diagnostic accuracy of ultrasonography for the detection of moderate to severe fatty livers in comparison to histology. The overall sensitivity and specificity levels for the ultrasound methods were 84.8% (95% CI, 79.5–88.9) and 93.6% (95% CI, 87.2–97.0), which was similar to that of other imaging methods (i.e., computed tomography and MRI). Our study showed that Hamaguchi’s score and the HRI demonstrated high diagnostic accuracy for the detection of steatosis (AUROC = 0.94). Furthermore, performance in terms of sensitivity, specificity, and Spearman’s coefficient (ρS) was good to excellent for the detection of steatosis (CAP ≥ 234 db/m) using optimal cut-off values. In regard to the detection of steatosis, the sensitivity and specificity level was 82.2% and 100.0% for Hamaguchi’s score and 86.9% and 94.8% for the HRI, respectively, and both methods achieved a high grading correlation with CAP (ρS = 0.79). These results are in agreement with previous studies that have validated these methods against LBs. Hamaguchi et al. [15] reported a 91.7% sensitivity and 100% specificity level for Hamaguchi’s score. Therefore, we conclude that Hamaguchi’s score has good performance for the detection of steatosis. However, the optimal thresholds to quantify all of its degrees has not been previously estimated. Therefore, based on our results, we propose new cut-off values, where a score of 0 points excludes steatosis, 1 or 2 points indicates low-grade steatosis (S1), 3 points suggests intermediate steatosis (S2), and a score of 4 points or greater indicates high-grade (S3) steatosis.

The HRI appeared to be a highly accurate modality to detect low and moderate steatosis, but our results indicate that it demonstrated poor sensitivity when distinguishing between moderate and severe steatosis. Thus far, only a few studies (based on small groups) have validated the HRI, and they have suggested different optimal cut-off values for steatosis grading. Webb et al. [16] were the first to describe a correlation between the HRI and LBs, with an AUROC of over 0.9 for all steatosis grades and 1.49 being the optimal cut-off value. Marshall et al. [21], in a study of 101 patients with biopsy-diagnosed NAFLD, reported an HRI sensitivity and specificity level of 100% and 54% respectively, with a cut-off value of 1.27. A similar optimal cut-off value of 1.24 was described by Borges et al. [22] on a small sample of 42 participants, with a sensitivity and specificity level of 93%. Chauhan et al. [23] estimated an optimal threshold of 2.01 to detect steatosis, with levels of sensitivity of 62.5% and specificity of 95.2%. According to the differences in the above-mentioned studies, it appears that further investigations are needed to establish optimal HRI cut-off values. In our study (based on AUROC results), we calculated new optimal thresholds for the HRI (S ≥ S1 1.41, S ≥ S2 1.56, S ≥ S3 2.015) (Table 3) and reported sensitivity and specificity levels, and Spearman’s coefficient for S ≥ S1, S ≥ S2, and S ≥ S3 equal to 91.6%, 86.2%, ρ = 0.78 and 94.0%, 80.2%, ρ = 0.75 and 57.6%, 90.6% and ρ = 0.52, respectively.

Except from these two evaluated in our study, several ultrasound-based techniques are available for the quantification of hepatic steatosis. The US-FLI score, which also estimates the presence of fatty sparing areas, the visualisation of the gall bladder wall, or the diaphragm, achieved an AUROC of 0.76 and 0.8 for the diagnosis of NASH and severe NASH, respectively [13]. Ultrasound-based techniques are recommended as the first-line procedures for imaging of NAFLD [14] and can be widely used for screening of other liver diseases, whereas CAP measurement only assesses hepatic steatosis and fibrosis.

#### 4.1.2. Biomarkers

In our study, the FLI and HSI performed similarly to what was originally described by Bedogni et al. [17] and Lee et al. [18]. In several studies comparing two of the biochemical scores tested in our study versus liver histology [24] or MRI, [25,26], the FLI and HSI performed equally well or slightly weaker to our results for the detection of liver steatosis. Koehler et al. [27], in their retrospective study on 2652 patients with ultrasound-detected NAFLD, reported an AUROC of 0.81 for the FLI. Good performance of the FLI for the diagnosis of NAFLD has also been confirmed in other studies, [12,17,28] including a report by Motamed et al. [29] that found an AUROC of 0.86 (95% CI: 0.85–0.87).

There are only a few published studies on the LAP, as originally described by Bedogni et al. [19]. Therefore, external validation for its clinical use is still needed. In a study on 101 polycystic ovary syndrome patients, Zheng et al. [30] reported that the LAP independently correlated with hepatic steatosis was established by CAP (ρ = 0.36, *p* < 0.001 vs. our result of ρ = 0.47). Lind et al. [25], in a newly published paper, estimated the diagnostic accuracy of the LAP at an AUROC of 0.77 (0.71–0.83). Our results yielded much better accuracy for the LAP, with AUROC equal to 0.92 (95% CI = 0.88–0.96, *p* < 0.001), but it achieved the lowest Spearman’s coefficient (ρ) at 0.57 in comparison to the FLI and HSI (ρ = 0.68 and 0.65, respectively). Despite a promising sensitivity level of 93.1%, the specificity of the LAP was estimated at only 62.1%. The sensitivity and specificity levels of the FLI and HSI were 77.8% and 86.2%, and 76.7% and 86.2%, respectively.

### 4.2. Strengths and Limitations of the Study

The strengths of this study include its prospective design, with well-defined participant characteristics with and without NAFLD and screening using standardised liver assessments to exclude patients with other causes of chronic liver disease, including excessive alcohol consumption. In addition, all participants underwent consecutive CAP and ultrasound assessments by the same certified operator, and biomarker evaluations occurred on the same day. To our knowledge, this is the first prospective study to assess the diagnostic accuracy of these five non-invasive methods in the general population in comparison to CAP, as well as the first to establish steatosis grading cut-off values for Hamaguchi’s score.

However, we acknowledge the following limitations of this study. First, LBs were not performed, as we found it unethical to perform them on patients with simple steatosis. LB is the reference method for the diagnosis of NAFLD, but is hindered by misdiagnosis and inaccuracies in identifying staging, which is partially due to the small sizes of tissue specimens (1/50,000 to 1/65,000 total volume) and the heterogeneous distribution of histological changes in the liver parenchyma [5,31]. Therefore, steatosis may be unevenly distributed, and sampling error remains a major challenge for LBs. Recently published literature indicates the excellent diagnostic value of MRI-PDFF in assessing liver steatosis, as well as quantifying the amount of liver fat. Most conducted studies on MRI-PDFF had relatively small numbers of patients so far, but the overall results are very promising [32]. MRI-PDFF appears to be a very accurate method for the assessment and follow-up of selected groups of patients, especially in clinical trials, but its cost and availability limit its use among large, unselected populations. Hence, we used a highly accurate, widely available, non-invasive quantitative modality, CAP, that has been validated against LBs and PDFF-MRI in numerous studies, [8,9,11] and against ultrasound [12]. It evaluates an area 100 times larger than LBs and has emerged as a novel biomarker for assessing hepatic steatosis. Second, in our study, we used both probes (M and XL), and probe selection was guided by an integrated tool according to skin-liver capsule distance. The CAP thresholds used were the same for both probes, as the literature has suggested there are no differences between measurements. However, in a recently published study by Caussy et al. [33], it was demonstrated that CAP values were significantly lower when obtained using the M probe as compared to the XL probe in the same participant, even when the probe was selected according to the participant’s BMI. Therefore, the authors concluded that different thresholds for the detection of NAFLD should be applied depending on the type of probe used for the CAP measurement. Moreover, along with the current study by Caussy et al., significantly different CAP thresholds for the diagnosis and staging of liver steatosis have been proposed over the past few years, depending on the reference method used [9,10,11,34]. These discrepancies could have affected our results, as we used the lowest cut-off values as opposed to the newly proposed ones. Had we used higher cut-off values, fewer subjects with disease would be diagnosed. Undoubtedly, the optimal cut-off values need to be validated using large cohorts for reliable diagnoses and staging of steatosis [35]. Currently, diagnostic confusion may seriously affect further clinical decisions, as well as the determination of the risk of NAFLD progression and its complications [35].

### 4.3. Implications for Clinical Use

In this study, we have demonstrated that all five non-invasive methods of steatosis detection (Hamaguchi’s score, HRI, FLI, HSI, and LAP) were able to discriminate between the presence and absence of steatosis, with an overall good diagnostic performance. Ultrasound-based techniques showed the highest accuracy in assessing liver steatosis of all evaluated non-invasive tests. Using a prospective study design, we showed that ultrasound assessment using Hamaguchi’s score or the HRI were also good diagnostic tools for the quantification of hepatosteatosis degrees. However, further cohort studies are needed to evaluate optimal diagnostic thresholds.

Our findings suggest that the above-mentioned tests can be useful screening tools for the detection of NAFLD, especially in patients with risk factors. PDFF-MRI and CAP remain relatively expensive and are not easily available. This is in contrast to ultrasound-based and biochemical diagnostic methods, which are possible to maintain in routine clinical practice. Our findings support the use of ultrasound as the imaging technique of choice for screening for NAFLD in the general population, especially given their low cost, non-invasive nature, wide availability, and the lack of radiation exposure involved. Moreover, simple biochemical algorithms may prove helpful in diagnosing steatohepatosis, particularly in the general practice setting, and in selecting patients who require further liver diagnostics.

## Figures and Tables

**Table 1:** Baseline characteristics of participants stratified by the presence of nonalcoholic fatty liver disease NAFLD (CAP ≥ 234 dB/m).

	Overall (*n* = 167)	NAFLD (*n* = 109)	Non-NAFLD (*n* = 58)	*p* Value
Demographic Data				
Age, years	52.6 (12.4)	53 (12.3)	51.9 (12.6)	0.58
Female	103 (62%)	66 (61%)	37 (64%)	0.68
Clinical Data				
Type 2 diabetes	16 (10%)	16 (15%)	0	<0.005
Hypertension	58 (35%)	46 (42%)	12 (21%)	<0.01
BMI, kg/m^2^	28.6 (5.1)	30.9 (4.4)	24.2 (2.9)	<0.0001
Waist circumference, cm	95.0 (15)	103.1 (10.6)	80.3 (9.2)	<0.0001
Hip circumference, cm	105.4 (9.5)	109.1 (9.4)	98.9 (5.4)	<0.0001
Statins	21 (13%)	20 (18%)	1 (2%)	<0.01
Obesity ^†^	116 (70%)	99 (90%)	17 (29%)	<0.0001
Laboratory measures				
AST, U/L	24 (12)	27 (14)	21 (7)	<0.001
ALT, U/L	23 (20)	26 (24)	17 (7)	<0.001
GGT, U/L	39 (32)	49 (35)	20 (14)	<0.0001
ALP, U/L	72 (25)	77 (26)	62 (19)	<0.001
Total bilirubin, mg/dL	0.5 (0.3)	0.5 (0.3)	0.5 (0.3)	0.37
Albumin, g/L	49 (4)	48 (3)	49 (6)	0.64
Glucose, mg/dL	100 (21)	103 (25)	95 (12)	0.21
Triglycerides, mg/dL	141 (75)	161 (82)	105 (40)	<0.0001
Total cholesterol, mg/dL	202 (42)	198 (43)	208 (40)	0.12
LDL-cholesterol, mg/dL	130 (37)	130 (38)	129 (36)	0.78
Ferritin, ng/mL	166 (164)	207 (186)	91 (69)	<0.0001
Fasting insulin, U/mL	17 (22)	20 (26)	11 (11)	0.01
HOMA-IR	3.8 (3.6)	4.5 (3.8)	2.7 (2.9)	<0.0001
Creatinine, mg/dL	0.9 (0.2)	0.9 (0.2)	0.9 (0.2)	0.43
Steatosis prediction algorithms				
FLI	59.2 (28.1)	73.7 (20.8)	34.6 (20.8)	<0.0001
HSI	36.9 (6.5)	39.9 (5.4)	31.6 (4.5)	<0.0001
LAP	57.4 (47.3)	76.3 (49.2)	24.6 (15.2)	<0.0001
Imaging Data				
Hamaguchi’s score	1.97 (2)	3 (1.7)	0 (0)	<0.0001
HRI	1.7 (0.5)	2 (0.4)	1.27 (0.2)	<0.0001
CAP	274 (60)	309.1 (42.8)	208.6 (19.3)	<0.0001
CAP in classes		S0: 58 (34.7%)	S1: 23 (13.8%) S2: 26 (15.6%) S3: 60 (35.9%)	<0.0001
IQR of CAP	26.6	26.9	26.3	0.60
TE	5.1 (2.1)	5.5 (2.4)	4.4 (1)	<0.001
IQR of TE	13.7	13.4	14.1	0.55
Probe size, *n*				
Use of M Probe	143 (85%)	84 (77%)	58 (100%)	<0.0001
Use of XL probe	25 (15%)	25 (23%)	0 (0%)	

^†^ Obesity was defined as BMI ≥ 30 kg/m^2^; Continuous variables are expressed as the mean (standard deviation, SD) unless otherwise noted as the *n* (%). The *p* value was determined by comparing patients with and without NAFLD using an independent-samples *t*-test, Mann-Whitney test, or a chi-square test, as appropriate. A *p*-value < 0.05 was considered significant. ALP, alkaline phosphatase; ALT, alanine aminotransferase; AST, aspartate aminotransferase; BMI, body mass index; CAP, controlled attenuation parameter; GGT, gamma-glutamyltransferase; FLI, fatty liver index; HIS, hepatic steatosis index; HRI, hepatorenal index; INR, international normalised ratio; IQR, interquartile range; LAP, lipid accumulation product; LDL, low-density lipoprotein; NAFLD, non-alcoholic fatty liver disease; TE, transient elastography.

**Table 2:** Diagnostic accuracy of Hamaguchi’s score, HRI, FLI, HSI, and LAP for the detection of hepatic steatosis.

	AUC (95% Confidence Interval)	Cut-Off Value	Sensitivity (%)	Specificity (%)	Kendall’s Tau-B	*p* Value
Detection of CAP ≥234 Hamaguchi’s score	0.94 (0.9–0.97)	2	82.2	100	0.79	<0.0001
Detection of CAP ≥234 HRI	0.94 (0.91–0.98)	1.49	86.9	94.8	0.79	<0.0001
Detection of CAP ≥234 FLI	0.90 (0.85–0.94)	60	77.8	86.2	0.62	<0.0001
Detection of CAP ≥234 HSI	0.90 (0.86–0.95)	36	76.7	86.2	0.61	<0.0001
Detection of CAP ≥234 LAP	0.92 (0.88–0.96)	23 ^a^ 30.5 ^b^	93.1	62.1	0.6	<0.0001

Kendall’s tau-b was used to evaluate the association between two variables measured on an ordinal scale. The *p* value was determined using chi-square tests. AUC, area under the curve; CAP, controlled attenuation parameter; HRI, hepatorenal index; HSI, hepatic steatosis index; FLI, fatty liver index; LAP, lipid accumulation product. ^a^ female, ^b^ male.

**Table 3:** Diagnostic accuracy of the new optimal thresholds of Hamaguchi’s score and HRI for grading of hepatic steatosis.

	Test Result Variable(s)	Cut-off Value	Sensitivity (%)	Specificity (%)	Kendall’s Tau-B	*p* Value
CAP ≥ S1	Hamaguchi ≥ S1	1	86.9	100	0.84	<0.0001
CAP ≥ S2	Hamaguchi ≥ S2	3	79.8	97.5	0.78	<0.0001
CAP ≥ S3	Hamaguchi ≥ S3	4	72.9	97.2	0.75	<0.0001
CAP ≥ S1	HRI ≥ S1	1.41	91.6	86.2	0.78	<0.0001
CAP ≥ S2	HRI ≥ S2	1.56	94	80.2	0.75	<0.0001
CAP ≥ S3	HRI ≥ S3	2.015	57.6	90.6	0.52	<0.0001

CAP grades S1–S3 are described according to Karlas et al. [10], with cut-off values of 234, 269, and 301 dB/m, respectively. Kendall’s tau-b was used to evaluate the association between two variables measured on an ordinal scale. The *p* value was determined using chi-square tests. CAP, controlled attenuation parameter; HRI, hepatorenal index.

**Table 4:** Optimal steatosis predicting models.

	Partial Correlation	*p* Value	R
Hamaguchi’s score	0.74	<0.0001	0.87
HSI	0.46	<0.0001	
Hamaguchi’s score	0.73	<0.0001	
BMI	0.29	<0.001	0.89
Ferritin	0.22	<0.01	
GGT	0.20	0.0149	
Hamaguchi’s score	0.74	<0.0001	0.91
LAP	0.17	0.06	
HSI	0.21	0.0192	

Multivariate forward and backward stepwise logistic regression analyses were used to evaluate algorithms that strongly correlated (R) with hepatic steatosis. CAP was used as the test variable. The scores and biochemical and anthropometric parameters were the independent variables. BMI, body mass index; CAP, controlled attenuation parameter; GGT, gamma-glutamyltransferase; HIS, hepatic steatosis index; LAP, lipid accumulation product.

## Data Availability

The data presented in this study are available on request from the corresponding author.

## Supplementary Materials

The following are available online at https://www.mdpi.com/article/10.3390/jcm10071507/s1, Table S1: STARD Checklist.
