# Supplementary material for: Diagnostic Accuracy of Non-Imaging and Ultrasound-Based Assessment of Hepatic Steatosis Using Controlled Attenuation Parameter (CAP) as Reference
Source: J Clin Med. 2021 Apr 4;10(7):1507. doi: 10.3390/jcm10071507 (PMC8038574; doi:10.3390/jcm10071507)
Supplement: Supplementary file 1 [file jcm-10-01507-s001.pdf]

SUPPLEMENTARY MATERIALS

SUPPLEMENTARY MATERIAL 1

Controlled attenuation parameter measurement

The examination was performed by trained physician by applying the probe through the intercostal spaces, in an anterior axillary line, at the level of xiphoid process of the sternum, to the right lobe of the liver, with the patient lying in a dorsal position with the right arm in maximal abduction. The final results of CAP and transient elastography were the median value of 10 measurements expressed in dB/m and kPa, respectively.

Hepatorenal Index measurement

To estimate the HRI, a visualization of the liver and right kidney in a single, longitudinal sonographic plane, usually at the intercostal space in the mid or anterior axillary line, was obtained. The regions of interest (ROI) were determined by the operator using circles in the most homogenous regions of the liver parenchyma and kidney cortex between the pyramids. This was done to avoid anatomic structures such as vessels, bile ducts or incidental cysts in the liver and the renal sinus, and perinephric fat in the kidney.[1] The HRI was then calculated automatically by the software incorporated in the ultrasound machine. Proper images and the HRI calculation were obtained for each patient three times. The final result was the average of three ratio measurements.

Hamaguchi Score measurement

Hamaguchi's ultrasound score uses four variables to assess liver steatosis. Hepatorenal echo contrast and liver parenchyma brightness are calculated together with score from 0 to 3. If they are negative (score 0) also the final summarised score is zero. Vessel blurring can be present

(score 1) and absent (score 0) and the attenuation depth scores from 0 to 2 points. Hepatic steatosis is defined by a score ≥2 and moderate/severe steatosis by score ≥4.[2]

REFERENCES:

1. Chauhan A, Sultan LR, Furth EE, Jones LP, Khungar V, Sehgal CM. Diagnostic accuracy of hepatorenal index in the detection and grading of hepatic steatosis: Factors Affecting the Accuracy Of HRI. J Clin Ultrasound 2016;44(9):580–6.
2. Hamaguchi M, Kojima T, Itoh Y, Harano Y, Fujii K, Nakajima T, et al. The Severity of Ultrasonographic Findings in Nonalcoholic Fatty Liver Disease Reflects the Metabolic Syndrome and Visceral Fat Accumulation. Am J Gastroenterol 2007;102(12):2708–15.

SUPPLEMENTARY TABLES

Supplementary Table S1. STARD Checklist

Section & Topic	No	Item	Reported on Page #
**TITLE OR ABSTRACT**			
	**1**	Identification as a study of diagnostic accuracy using at least one measure of accuracy (such as sensitivity, specificity, predictive values, or AUC)	1
**ABSTRACT**			
	**2**	Structured summary of study design, methods, results, and conclusions (for specific guidance, see STARD for Abstracts)	1
**INTRODUCTION**			
	**3**	Scientific and clinical background, including the intended use and clinical role of the index test	2-3
	**4**	Study objectives and hypotheses	2-3
**METHODS**			

	**5**	Whether data collection was planned before the index test and reference standard were performed (prospective study) or after (retrospective study)	4
Study design			
Participants	**6**	Eligibility criteria	4
	**7**	On what basis potentially eligible participants were identified (such as symptoms, results from previous tests, inclusion in registry)	4
	**8**	Where and when potentially eligible participants were identified (setting, location and dates)	4
	**9**	Whether participants formed a consecutive, random or convenience series	4
Test methods	**10a**	Index test, in sufficient detail to allow replication	5-6
	**10b**	Reference standard, in sufficient detail to allow replication	5
	**11**	Rationale for choosing the reference standard (if alternatives exist)	5
	**12a**	Definition of and rationale for test positivity cut-offs or result categories of the index test, distinguishing pre-specified from exploratory	5
	**12b**	Definition of and rationale for test positivity cut-offs or result categories of the reference standard, distinguishing pre-specified from exploratory	5-6
	**13a**	Whether clinical information and reference standard results were available to the performers/readers of the index test	
	**13b**	Whether clinical information and index test results were available to the assessors of the reference standard	
Analysis	**14**	Methods for estimating or comparing measures of diagnostic accuracy	7

	**15**	How indeterminate index test or reference standard results were handled	7
	**16**	How missing data on the index test and reference standard were handled	
	**17**	Any analyses of variability in diagnostic accuracy, distinguishing pre-specified from exploratory	7
	**18**	Intended sample size and how it was determined	
**RESULTS**			
Participants	**19**	Flow of participants, using a diagram	Suppl. Figure 1
	**20**	Baseline demographic and clinical characteristics of participants	8 and Table 1
	**21a**	Distribution of severity of disease in those with the target condition	8 and Table 1
	**21b**	Distribution of alternative diagnoses in those without the target condition	8 and Table 1
	**22**	Time interval and any clinical interventions between index test and reference standard	5
Test results	**23**	Cross tabulation of the index test results (or their distribution) by the results of the reference standard	8-9
	**24**	Estimates of diagnostic accuracy and their precision (such as 95% confidence intervals)	8-9
	**25**	Any adverse events from performing the index test or the reference standard	NA
**DISCUSSION**			
	**26**	Study limitations, including sources of potential bias, statistical uncertainty, and generalisability	12-13
	**27**	Implications for practice, including the intended use and clinical role of the index test	14
**OTHER INFORMATION**			
	**28**	Registration number and name of registry	5

	**29**	Where the full study protocol can be accessed	Upon request
	**30**	Sources of funding and other support; role of funders	Title page
